# Exploiting Amoeboid and Non-Vertebrate Animal Model Systems to Study the Virulence of Human Pathogenic Fungi

**DOI:** 10.1371/journal.ppat.0030101

**Published:** 2007-07-27

**Authors:** Eleftherios Mylonakis, Arturo Casadevall, Frederick M Ausubel

**Affiliations:** University of British Columbia, Canada

## Abstract

Experiments with insects, protozoa, nematodes, and slime molds have recently come to the forefront in the study of host–fungal interactions. Many of the virulence factors required for pathogenicity in mammals are also important for fungal survival during interactions with non-vertebrate hosts, suggesting that fungal virulence may have evolved, and been maintained, as a countermeasure to environmental predation by amoebae and nematodes and other small non-vertebrates that feed on microorganisms. Host innate immune responses are also broadly conserved across many phyla. The study of the interaction between invertebrate model hosts and pathogenic fungi therefore provides insights into the mechanisms underlying pathogen virulence and host immunity, and complements the use of mammalian models by enabling whole-animal high throughput infection assays. This review aims to assist researchers in identifying appropriate invertebrate systems for the study of particular aspects of fungal pathogenesis.

## Introduction

Most studies of bacterial or fungal infectious diseases focus separately on the pathogenic microbe, the host response, or the characterization of therapeutic compounds. Compartmentalization of pathogenesis-related research into an analysis of the “pathogen”, the “host,” or the “antimicrobial compound” has largely been dictated by the lack of model systems in which all of these approaches can be used simultaneously, and by the traditional view that microbiology, immunology, and chemical biology and pharmacology are separate disciplines. The arbitrary separation of these fields is no longer necessary, as genetic and genomic tools for a number of pathogenic microbes are now available and an extensive understanding of virulence mechanisms and host responses has been achieved. We see the traditional separation of these disciplines as a major hindrance to the development of novel antimicrobial agents and groundbreaking therapies. For example, we think that there are a number of shortcomings with the currently accepted “gold standard” approach of using an in vitro assay to determine the minimal inhibitory concentration of compounds against a pathogen of interest. Measuring minimal inhibitory concentrations does not allow the simultaneous evaluation of toxicity or the identification of compounds that have an immunomodulatory effect that augments the host response to the infection or those that have a dual effect (a direct antimicrobial effect as well as an immunomodulatory effect).

A number of different invertebrate host model systems have been described in the past few years (Box 1) that allow multidisciplinary studies of host–fungal interactions from the perspectives of both the pathogen and the host. A variety of fungi involved in mammalian pathogenesis can infect and kill non-vertebrate model hosts. Consequently, many researchers have turned to non-vertebrates as facile, ethically expedient, relatively simple, and inexpensive hosts to model a variety of human infectious diseases. An important advantage of many non-vertebrate hosts is that they are small enough to fit in microtiter plates, which makes it possible to use them in high throughput studies designed to scan pathogen genomes for virulence-related genes or to scan chemical libraries for antimicrobial compounds. Moreover, because many non-vertebrate hosts are genetically tractable, they can be used in conjunction with an appropriate pathogen to study host innate immunity. As it has become apparent that it is important to select the model host that is best suited to test a specific hypothesis (Table 1), this review is designed to help investigators in the field of fungal pathogenesis address some important questions as they navigate through the advantages and disadvantages of different non-vertebrate models.

**Table 1:**
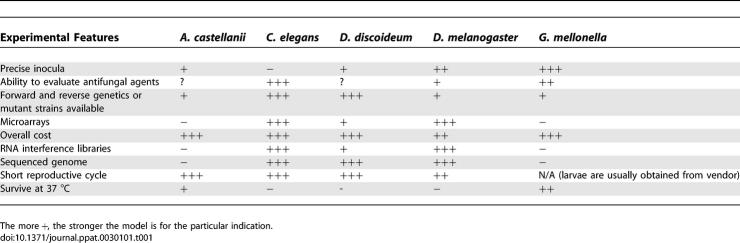
Comparison of Representative Invertabrate Model Hosts That Have Been Used for the Study of Fungal Pathogenesis

## What Is the Virulence Trait under Study?

A recurrent finding in recent studies of fungal virulence factors is that many of the same pathogenesis traits are required for virulence in both mammals and non-vertebrate hosts. For example, in the pathogenic yeast *Cryptococcus neoformans,* genes associated with the *GPA1, PKA1, PKR1,* and *RAS1* signal transduction pathways, which regulate important virulence factors in mammalian pathogenesis, have also been shown to play a role in the killing of the nematode Caenorhabditis elegans and the insects Drosophila melanogaster and Galleria mellonella [1–3]. The extent of the similarities between fungal virulence factors required in mammalian and non-vertebrate hosts is illustrated by studies of the C. neoformans mating locus. More specifically, the role of the C. neoformans mating locus in invertebrates is similar to that in mice [4–6]. The *MFa1* gene, which regulates production of the mating type MFα pheromone and is associated with increased virulence in mammalian models [5], is involved in the killing of nematodes [2] and insects [3] by C. neoformans var. *neoformans,* but not by C. neoformans var. *grubii* [3,4].

The similarities between simple model hosts and mammals extend in some cases to the intracellular fate of fungi. C. neoformans replicates inside macrophages as well as within the amoeba *Acanthamoeba castellani,* and in both cases, the ingested C. neoformans synthesize capsular polysaccharides that are required for virulence [7–11]. It is noteworthy that stimulation of A. castellani with arachidonic acid and prostaglandins enhances the phagocytic efficacy of the amoeboid cells [12], an observation that is relevant to amoeba–fungal interactions given that many fungal species produce these compounds [13,14]. However, further study is needed, as the intracellular fate of cryptococci in insect hemocytes is not known.

**Box 1.**

Methodology Used to Study Fungal Pathogenesis in Representative Invertebrate Model Hosts

**Drosophila:**

- Injection: pricking the fly thorax, or abdomen, with a needle that has been dipped in a suspension of the microbe. If administration of exact inocula is necessary, microinjection of a precise dose of microbes directly into the body cavity is available.
- “Natural infection”
   i. Feeding *Drosophila* larvae or adults with a concentrated solution that has been mixed with their food,
   ii. spraying fungal spores directly onto the fly exoskeleton, or,
   iii. “rolling” flies on agar plates that have been spread with the pathogen (“rolling assay”).

**Acanthamoeba:**

- Co-incubation of amoebae and C. neoformans leads to phagocytosis, amoebae killing, or fungal cell killing, depending on the species of amoebae or cryptococcal strain.

**C. elegans:**

- Killing assays involve transferring C. elegans animals (usually at the L4 developmental stage) from a lawn of Escherichia coli strain OP50 to a lawn of the pathogen.

**D. discoideum:**

- Co-incubation of amoeboid cells of D. discoideum and C. neoformans leads to phagocytosis, although the process is less efficient than it is with amoebae, possibly because of the smaller size of the host cell.

**G. mellonella:**

- A syringe is used to inject aliquots of the inoculum into the hemocoel of G. mellonella caterpillars in the final instar larval stage via a larva proleg.

An exciting hypothesis based on the conservation of fungal virulence factors for diverse hosts is that fungal signaling cascades and associated virulence factors that confer a survival advantage during the infection of mammalian hosts originally evolved during the interaction of fungi with environmental predators [15], including insects such as *D. melanogaster,* that primarily consume plant saprophytic or pathogenic fungi. Similarly, many bacterial virulence factors, especially in opportunistic environmental pathogens such as *Pseudomonas aeruginosa,* are involved in both mammalian pathogenesis and predation avoidance by nematodes and amoebae [16–18].

The phenomenon of fungal dimorphism, an important aspect of fungal virulence in mammals, may also have emerged as a mechanism for escaping predators. For example, Histoplasma capsulatum transitions to a hyphal form when exposed to A. castellani amoebae [19] and Candida albicans filaments upon ingestion by C. elegans [20]. These findings complement an earlier observation that C. neoformans hyphal forms survive predation by *Acanthamoebae polyphaga,* whereas yeast forms are consumed [8]. In vitro, these transitions to hyphal forms only occur at 37 °C, but they can occur at ambient temperatures in the presence of the invertebrate hosts. Given that there are many thousands of species that are microherbivores, it is likely that the ability of fungi to interconvert between yeast and hyphal forms provides protection against particular predators.

In addition to the potential evolutionary origins of fungal virulence, interactions between fungi and invertebrate hosts may play an important role in the maintenance of virulence in mammalian hosts. For example, passage of an avirulent H. capsulatum strain in amoeba is associated with an increase in virulence for mice [19]. Also, the C. neoformans Ras signaling cascade is required for cryptococcal virulence because it regulates growth at high temperature (37 °C) [21]. However, *RAS1* plays a significant role in C. elegans ([2] and E. Mylonakis, unpublished data), *Drosophila* [1], and G. mellonella [3] pathogenesis at 20 °C, 25 °C, and 30 °C, respectively.

**Figure 1:**
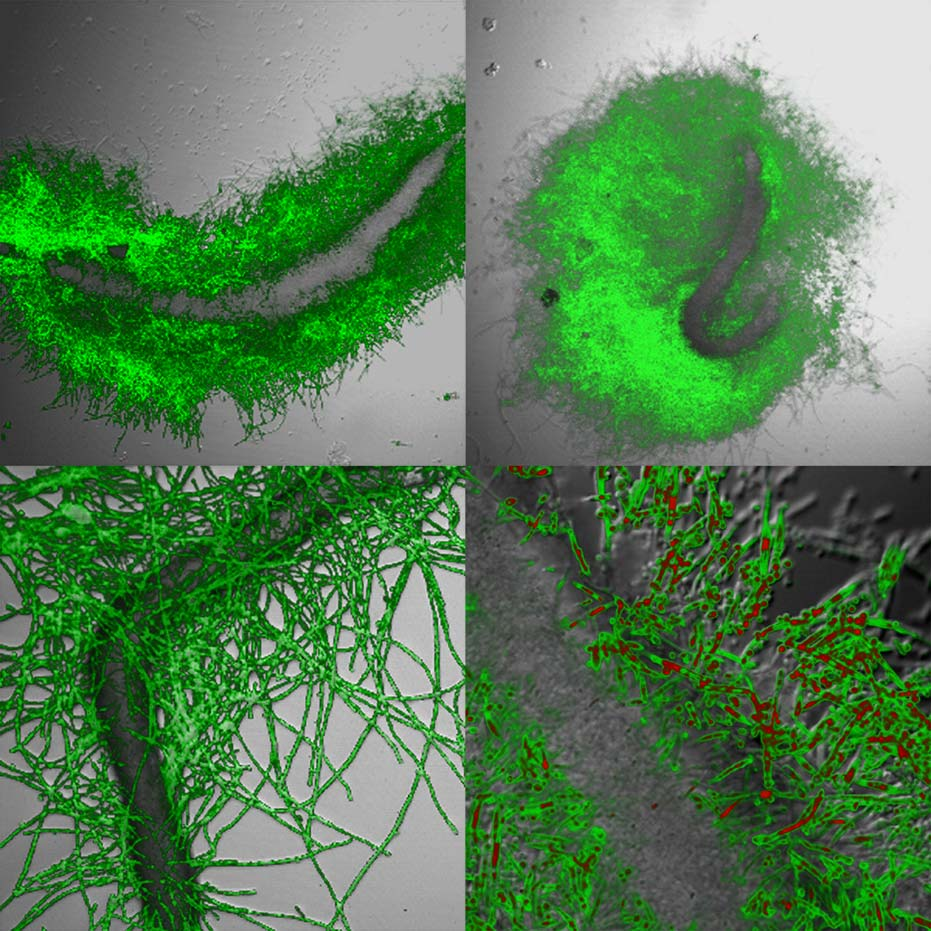
Dead C. elegans Nematodes Infected by C. albicans Filamentation is instrumental for *Candida* virulence in mammals and is also involved in the killing of C. elegans [20]. The four panels show consequences of infecting *C. elegans glp-4;sek-1* animals with C. albicans and then moving them into pathogen-free liquid medium. The top panels show that C. albicans cells persist within the C. elegans intestine and form hyphae (green) that break through the C. elegans cuticle, leaving a C. elegans “ghost” (dark structure) that outlines where the cuticle used to be. The bottom panels show that *Candida* cells develop filaments (green) that differentiate into hyphae, long continuous germ tubes separated by true septin rings, or pseudohyphae, chains of distinct cells that fail to separate. Pictures were taken with a confocal laser microscope (TCS NT; Leica Microsystems, http://www.leica-microsystems.com/). Concanavalin A-Alexafluor (fluorescence emission at 519 nm) is a fluorescent green dye that binds to polysaccharides. FUN-1, which was also used in the bottom right panel, is a fluorescent yellow dye that is absorbed by metabolically active fungal cells and fluoresces red when illuminated with a fluorescence emission 480 nm [20].

Importantly, there is a significant amount of host specificity between fungal pathogens and non-vertebrate hosts that needs to be taken into account when selecting an invertebrate system. For example, the dimorphic fungi *Blastomyces dermatidis, Sporothrix schenckii,* and *H. capsulatum,* as well as the basidiomycetous fungus *C. neoformans,* kill A. castellanii and grow in the presence of the amoebae, whereas A. castellani readily kills C. albicans [19]. Also, the experimental conditions must be considered, as they should be conducive to the virulence factor under study. For example, C. albicans filamentation is only induced within C. elegans when the nematode is in liquid medium [20] (Figure 1).

**Figure 2:**
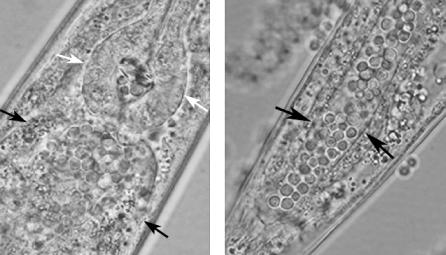
Wild-Type C. neoformans Accumulates in the Gastrointestinal Tract Intact yeast cells are present in the distended (A) proximal and (B) distal gastrointestinal tract of C. elegans after feeding for 36 h on C. neoformans strain KN99α. Black arrows point to the intestinal lumen. The white arrowheads note the pharyngeal grinder organ, which functions to disrupt ingested organisms.

Interestingly, in many cases, non-vertebrate hosts allow comparative studies of fungal pathogenesis. For example, C. neoformans and *C. albicans,* which have very different ecological niches, kill C. elegans nematodes by implementing significantly different pathogenic processes. The mechanism by which cryptococcal cells kill C. elegans is not clear. They do not adhere to the nematode intestine, and nematodes exposed to cryptococcal lawns are able to defecate the cryptococcal cells upon transfer to liquid medium, thereby clearing the cryptococcal infection [2,20]. In contrast, *Candida* species establish persistent infections of the C. elegans intestine, dissolve nematode tissues, and break through the nematode cuticle by forming an impressive network of filaments [20] (Figure 2).

Another important point is that even when a fungal species is virulent in several hosts, different virulence traits are not necessarily equally important in the different systems. For example, the cryptococcal polysaccharide capsule that is essential for mammalian infection also enables cryptococci to survive phagocytosis by A. castellanii [11] or Dictyostelium discoideum [15] and to kill the insect G. mellonella [3]. However, acapsular C. neoformans strains are still able to kill C. elegans [2].

## What Is the Desired Host Response?

In addition to the significance of the virulence trait under study, consideration should be given to the host response. Host innate immune responses are broadly conserved across many host phyla, and fungal virulence factors may target signal transduction cascades that are shared between mammals and non-vertebrate environmental predators. Evolutionary conservation of innate mechanisms of host defense is exemplified by the conservation of the Toll-like signaling pathways in mammals and insects. Since Lemaitre et al. demonstrated that the Toll receptor, previously known for its essential role during *Drosophila* embryonic development, is required for antifungal defense in *Drosophila,* and that mutations in the Toll signaling pathway dramatically reduced survival after Aspergillus fumigatus infection [22], *Drosophila* has emerged as a preferred animal host in which to study the genetic control of immune recognition and response. *Drosophila* provides genetic tractability and a variety of genomic tools, including full-genome microarrays [23,24] and RNA interference libraries [25,26], that can be utilized to identify immune-related genes. An advantage of *Drosophila* is that both systemic and local inoculations can be performed (Box 1), because the infection can be achieved by systemic injection as well as by local infection, such as feeding. However, it should be noted that the mechanical manipulation associated with systemic inoculation in *Drosophila* might affect the humoral [27–29] and cellular [30] responses, and this should be taken into account when evaluating the response of *Drosophila* to fungi, since such a response may be due to the trauma of the inoculation and not the fungal infection.

Genetic screens in *Drosophila* used to isolate mutants unable to induce humoral responses to infection led to the identification of components of two parallel signaling cascades, the Toll and Imd pathways [31], both of which contribute to the *Drosophila* defense response against microbes [32,33]. Although there appear to be some exceptions, the Toll pathway is primarily activated by fungal and Gram-positive bacterial pathogens, whereas the Imd pathway is primarily activated by Gram-negative bacteria. A major area of focus has been the identification of the pathogen recognition receptors that function in these two *Drosophila* immune pathways. This effort led to the discovery of families of peptidoglycan binding proteins and Gram-negative binding proteins (GNBPs) [34–37]. Recently published work shows that the GNBP family of pathogen recognition proteins, in particular GNBP3, is also required for the activation of the Toll pathway in response to fungal pathogens [37].

In contrast to *Drosophila,* mammals have at least ten members of the Toll-like receptor (TLR) family that participate in pathogen recognition [38,39]. TLRs are transmembrane proteins that consist of an extracellular leucine-rich repeat domain and an intracellular Toll–interleukin 1 receptor (TIR) domain. In both *Drosophila* and mammals, a key signaling component downstream of TLRs is the TIR domain that contains protein MyD88, which in mammals is essential for inflammatory cytokine production through all TLRs. In mammals, as in *Drosophila,* TLRs and MyD88 have been implicated in the pathway involved in recognition of fungal pathogens, including A. fumigatus [39–41], C. albicans [41,42], Coccidioides posadasi [43], C. neoformans [44,45], and *Pneumocystis* [46]. The role of individual receptors such as TLR2, TLR4, and TLR9 in MyD88 activation varies depending on the fungus and the site of infection [38].

The similarities between insect and human responses to pathogenic fungi are not limited to D. melanogaster. For example, the host response of the greater wax moth, *G. mellonella,* includes six types of hemocytes, phagocytosis, and “nodulation” (encapsulation of large invading pathogens by layers of hemocytes) (reviewed in [47]). Hemocytes of G. mellonella are capable of phagocytosing fungal cells of C. albicans [48], C. neoformans [3], and Aspergillus spp. [49,50], and the kinetics of phagocytosis and microbial killing are similar to those of human neutrophils [48]. Interestingly, immunoblotting of G. mellonella hemocytes with antibodies raised against human neutrophil phox proteins revealed the presence of proteins homologous to p67phox and p47phox [48] that, in humans, has been associated with chronic granulomatous disease [51].

Although the Toll pathway is conserved between insects and mammals, the complete pathway does not appear to be required for an effective immune response in all non-vertebrates. *C. elegans,* for example, has structural homologs of some Toll pathway components, including *tol-1, trf-1, pik-1,* and *ikb-1,* which are homologs of mammalian TLRs, TRAF6, IRAK, and IkB, respectively, but is missing homologs of MyD88 and NF-κB [52]. On the other hand, C. elegans does have a TIR domain protein, referred to as TIR-1, that is a homolog of the human SARM protein [53,54]. In *C. elegans,* however, TIR-1 functions as a positive regulator of the antimicrobial peptide NLP-31, a member of the neuropeptide-like protein family. Purified NLP-31 has antifungal activity towards *Drechmeria coniospora, Neurospora crassa,* and *A. fumigatus,* and C. elegans is more susceptible to D. coniospora when *tir-1* is silenced via RNA interference [54]. As in *Drosophila,* genetic screens have also been carried out to identify components of a presumptive C. elegans innate immune response pathway upstream of induced defense responses. A forward genetic screen demonstrated a requirement for a conserved p38 mitogen-activated protein kinase pathway in C. elegans immunity [55] that functions downstream of TIR-1 [53,56].

It is reasonable to expect that interaction of pathogens with evolutionarily distant hosts will continue to provide useful insights into the study of the evolution of immune signaling and to enhance our understanding of evolution in general. For example, studying the commonalities in innate immunity cascades sheds light on whether coelomates form a single clade, the Coelomata, or whether all animals that molt an exoskeleton (arthropods and nematodes) form a distinct clade, the Ecdysozoa [57–59]. For example, the presence of highly conserved Toll signaling pathways in *Drosophila* and mammals and the lack of key Toll signaling components in C. elegans may argue in favor of the Coelomata hypothesis [59].

## What Is the Appropriate Endpoint of a Pathogenicity Assay?

Many microbial virulence traits are induced only in the host, and therefore the study of these traits may require detection in vivo. The molecular mechanisms by which pathogenic microbes interact with human hosts are most commonly studied using mammalian models of infection. However, the study of pathogenesis in mammalian models is complicated by difficulties of handling, long reproductive cycles, small brood sizes, physiological and anatomical complexity, regulatory requirements, high cost, and ethical considerations. In particular, the use of invertebrate hosts in genetic screens that involve a large number of host individuals is especially appealing. In invertebrate model hosts, the most common phenotype used to monitor the progress of an infection is the death of the host. In one such study, the killing of C. elegans by C. neoformans was used to screen a library of random C. neoformans insertion mutants. Approximately 2% of the mutants tested demonstrated attenuated virulence in *C. elegans,* and these phenotypes were verified by showing that they persisted after crossing the relevant mutations back into a wild-type strain [60].

Mutated invertebrate hosts that express a convenient reporter or are immunocompromised are particularly useful when there is a need for an unambiguous endpoint. For example, as noted above, injection of fungi into wild-type *Drosophila* leads to an increase of antimicrobial peptides that are very effective against fungi [22,33,61–63]. The increase in antimicrobial peptides is not observed in Toll mutants of *Drosophila* that are susceptible to systemic inoculation, and researchers have demonstrated that Toll mutants are susceptible to A. fumigatus [22], C. neoformans [1], or Candida spp. [61] pathogenesis. In addition to facilitating experiments, these studies also provide an interesting example of microbial opportunism. In humans, invasive fungal infections are most commonly associated with immune deficiency of the host. This phenomenon of microbial opportunism (the fact that low virulence microbes can cause disease in hosts with impaired immunity) appears to have counterparts at the unicellular level. For example, acapsular C. neoformans are not able to grow in wild-type cells of the slime mold *D. discoideum,* but D. discoideum mutants defective in Myosin VII (which is involved in cell and particle adhesion during phagocytosis) are susceptible to acapsular C. neoformans [15].

Death of the host is not the only phenotype that has been used to monitor pathogenesis in non-vertebrate hosts. C. neoformans not only kills *C. elegans,* but also accumulates to high levels in the C. elegans intestinal tract and prevents the self-fertilizing hermaphrodites from producing a brood of progeny. In contrast to *C. neoformans,* nonpathogenic cryptococci such as Cryptococcus laurentii are unable to survive ingestion by the nematode, and do not interfere with progeny production. By screening a library of C. neoformans randomly generated insertional mutants for strains that permitted the production of C. elegans progeny, researchers have identified mutants that had a progeny-permissive phenotype. These mutants corresponded to genes involved in maintenance of the cryptococcal cell wall, including a homolog of *Saccharomyces cerevisiae ROM2,* which encodes a Rho1p activator in the protein kinase C pathway that regulates cell wall integrity [64]. Interestingly, *C. neoformans rom2* was unable to survive ingestion by the nematode and is avirulent in an inhalation infection model in mice [64].

The use of invertebrates in automated, high throughput in vivo assays can be viewed as an emerging technology related to the use of invertebrate hosts. An example is a whole-animal C. elegans assay that allows screening for low molecular weight compounds with activity against C. albicans. *C. albicans,* as well as other Candida spp., are ingested by C. elegans and establish a persistent lethal infection in the C. elegans intestinal tract. Importantly, key components of *Candida* pathogenesis in mammals, such as biofilm and filament formation, are also involved in nematode killing [20]. A liquid assay for *C. elegans–C. albicans* pathogenesis was developed using standard 96-well or 384-well microtiter plates, which hold about 25 or 15 worms per well, respectively. A pilot screen of 1,266 compounds with known pharmaceutical activities identified 15 (∼1.2%) that prolonged survival of C. albicans–infected nematodes and inhibited in vivo filamentation of C. albicans. Three of the 15 compounds have been tested in mice, and two out of three of these compounds, caffeic acid phenethyl ester, a major active component of honeybee propolis, and the fluoroquinolone agent enoxacin, exhibited anti-fungal activity in mice [20].

## Selecting an Invertebrate Model System

No invertebrate model system reproduces all aspects of mammalian infection, and any particular invertebrate is likely to have specific advantages, including the fact that the selected host may be found in the natural environment of the fungal pathogen in question. The selection of a model system for studying fungal virulence is largely dependent on the specific pathogen virulence-related factors, the specific host innate immune responses of interest, and the scientific question asked. If the goal is to study innate immune responses, the choice most likely will require the selection of a multicellular model genetic organism such as *Drosophila* or C. elegans. If the goal is to study phagocytosis and/or the outcome of ingestion, the choices include unicellular organisms such as amoebae and slime mold or invertebrates such as insects with phagocytic cells. Similarly, if the goal is to study fungal processes that are operative at mammalian temperatures, then one must select a model system that is thermotolerant, such as G. mellonella or amoebae, or the emerging model Panagrellus redivivus that can be propagated at 37 °C [65].

The identification of processes that are reproduced in more than one host may represent ancient mechanisms of cell–cell interactions. Nevertheless, it is important to keep in mind when choosing a model host that metazoans, protista, and slime molds are separated by enormous evolutionary distances and that many host-specific phenomena are likely to exist. For example, recent work suggests that internalization of C. neoformans cells by alveolar macrophages increases the dissemination of C. neoformans to the central nervous system [66], which would be difficult to study in an invertebrate model.

## Conclusions

In summary, workers in the field of fungal pathogenesis have the opportunity to select from several invertebrate animal model systems in their studies. An understanding of the unique strengths and limitations associated with each model host is necessary, as particular virulence traits are not equally important in all systems and genetic tractability is not available in all model hosts. Although these model systems are currently available for studying fungal pathogenicity in non-mammalian hosts, it is important to consider that they represent a minute fraction of the potential hosts available. Among the protista, for example, only a couple of amoebae species have been studied in a kingdom that includes a large number of species. Consequently, better model systems may be identified in the future, and there is a need to continue to explore fungal interactions with non-mammalian hosts. Nevertheless, the systems currently available provide investigators with many new options for studying fungal virulence and pathogenicity. Selecting the model that best addresses an experimental hypothesis is dependent on the questions asked and the strengths and limitations of the various systems. Invertebrate model systems have already provided novel insights into the origins of fungal pathogenicity, and one can confidently expect that they will continue to facilitate the study of the evolution and maintenance of fungal virulence.

## Abbreviations

GNBP: Gram-negative binding protein
TIR: Toll–interleukin 1 receptor
TLR: Toll-like receptor
